# Optimizing Nutritional Decisions: A Particle Swarm Optimization–Simulated Annealing-Enhanced Analytic Hierarchy Process Approach for Personalized Meal Planning

**DOI:** 10.3390/nu16183117

**Published:** 2024-09-15

**Authors:** Fatemeh Sarani Rad, Maryam Amiri, Juan Li

**Affiliations:** Department of Computer Science, North Dakota State University, Fargo, ND 58105, USA; fatemeh.saranirad@ndsu.edu (F.S.R.); m.amiri@ndsu.edu (M.A.)

**Keywords:** multi-criteria decision-making, analytic hierarchy process, meal planning, particle swarm optimization, simulated annealing, nutritional counseling, personalized nutrition

## Abstract

Background/Objective: Nutritionists play a crucial role in guiding individuals toward healthier lifestyles through personalized meal planning; however, this task involves navigating a complex web of factors, including health conditions, dietary restrictions, cultural preferences, and socioeconomic constraints. The Analytic Hierarchy Process (AHP) offers a valuable framework for structuring these multi-faceted decisions but inconsistencies can hinder its effectiveness in pairwise comparisons. Methods: This paper proposes a novel hybrid Particle Swarm Optimization–Simulated Annealing (PSO-SA) algorithm to refine inconsistent AHP weight matrices, ensuring a consistent and accurate representation of the nutritionist’s expertise and client preferences. Our approach merges PSO’s global search capabilities with SA’s local search precision, striking an optimal balance between exploration and exploitation. Results: We demonstrate the practical utility of our algorithm through real-world use cases involving personalized meal planning for individuals with specific dietary needs and preferences. Results showcase the algorithm’s efficiency in achieving consistency and surpassing standard PSO accuracy. Conclusion: By integrating the PSO-SA algorithm into a mobile app, we empower nutritionists with an advanced decision-making tool for creating tailored meal plans that promote healthier dietary choices and improved client outcomes. This research represents a significant advancement in multi-criteria decision-making for nutrition, offering a robust solution to the inconsistency challenge in AHP and paving the way for more effective and personalized dietary interventions.

## 1. Introduction

Effective meal planning is a cornerstone of nutritional counseling, serving as a personalized roadmap toward achieving an individual’s health and wellness goals. However, the process of crafting optimal meal plans is far from straightforward. Nutritionists must navigate a complex landscape of competing factors, including nutritional value, adherence to dietary restrictions or preferences (e.g., vegetarian, vegan, and gluten-free), cultural considerations, budgetary limitations, palatability, and even sustainability concerns. This multi-faceted decision-making process requires a structured approach to ensure that all relevant criteria are considered and prioritized appropriately.

Multi-criteria decision-making (MCDM) methods, such as the Analytic Hierarchy Process (AHP), have emerged as valuable tools in this context [1]. AHP, introduced by Saaty in 1980 [2], enables nutritionists to systematically break down complex meal-planning decisions into a hierarchical structure of goals, criteria, sub-criteria, and alternative meal options [3,4]. This structured approach facilitates a comprehensive evaluation of diverse factors, enabling nutritionists to tailor meal plans to individual needs and preferences.

However, the efficacy of AHP hinges upon the consistency of pairwise comparisons used to establish the relative importance of different criteria. Inconsistencies within the weight matrix—a common issue in AHP—can result in skewed decision-making, undermining the method’s effectiveness [5]. This challenge is particularly pronounced in nutrition, where the accurate prioritization of dietary criteria is paramount for achieving desired health and preference outcomes.

Recent advancements in the field have sought to address the consistency problem through various algorithms and optimization methods [5,6,7]. Yet, these solutions often face limitations, such as increased computational demands or susceptibility to local optima, which can hinder their practical application [8,9]. Moreover, existing approaches may not fully capture the intricate interplay of biological, socioeconomic, and cultural factors that shape individual dietary choices, as highlighted in our previous work on personalized meal planning for diabetic patients [4].

Recognizing these constraints, our work introduces a novel Particle Swarm Optimization–Simulated Annealing (PSO-SA) hybrid algorithm that synergizes both methods. This innovative algorithm aims to refine the initial, inconsistent matrix into a consistent one, thereby enhancing the decision-making process for nutritionists. By integrating PSO’s global search capabilities with SA’s local search precision, we optimize the balance between exploration and exploitation, ensuring a thorough and efficient search for the most consistent and accurate representation of the nutritionist’s expertise and the client’s unique needs and preferences.

The significance of this research lies not only in its potential to revolutionize the way nutritionists prioritize criteria for meal selection—a task that directly impacts meal planning and dietary recommendations—but also in its ability to bridge the gap between theoretical models and practical applications. By ensuring the consistency and accuracy of the decision matrix, our algorithm promises to elevate the standard of nutritional guidance provided to individuals, fostering improved health outcomes and greater client satisfaction.

## 2. Related Works

Optimizing meal planning is a complex task that extends beyond nutritional considerations, encompassing personal preferences, cultural influences, and economic factors.

There has been a concerted effort in the literature to explore personalized meal planning, particularly for diabetic patients. The research emphasizes the importance of considering a variety of factors, including taste preferences, nutritional content, budgetary constraints, and health requirements, to effectively manage diabetes [4,10].

Recent technological advancements have led to the development of AI-powered meal planners that consider health concerns, nutritional needs, and personal preferences—for example, Amiri et al. [11] developed a system that uses reinforcement learning to create meal plans with high user acceptance. Othman et al. [12] designed a recommender system for diabetic patients incorporating blood glucose readings and BMI.

Zioutos et al. [13] introduced a recommendation system that offers personalized meal plans by leveraging collaborative filtering and individuals’ health history analysis. This system’s ability to dynamically adapt to users’ constraints and preferences provides a novel approach to meal plan personalization.

Azzimani et al. [14] proposed an AI-based approach for personalized nutrition and food menu planning, utilizing machine learning algorithms for practical solutions in the nutrition field. Brintha et al. [15] developed a food recommendation system for predictive diabetic patients using Artificial Neural Networks (ANN) and Convolutional Neural Networks (CNN), providing a food recognition and tracking system on their website. Rastogi et al. [16] applied learning and semantics to offer personalized food recommendations, utilizing a health coach platform that recommends personalized selections of food recipes to diabetic patients.

Significant advancements have been made in meal planning and MCDM, for instance, Simpson et al. [17] provided a framework for identifying nutritional targets, while Gazan et al. [18] emphasized the role of mathematical optimization in developing sustainable diets. Srdjevic [19] improved the analytic hierarchy process by incorporating various prioritization methods. However, Field [20] identified challenges in implementing multisectoral nutrition planning, underlining the need for high-level political support. Lakshmi et al. [21] revolutionized personalized nutrition using the Fuzzy Analytic Hierarchy Process (F-AHP), the Fuzzy Technique for Order of Preference by Similarity to Ideal Solution (Fuzzy TOPSIS), and Multi-criteria Selection Analysis, involving dieticians and medical professionals in the dietary plan evaluation process. These studies collectively highlight the complexity of meal planning and the necessity to consider a wide range of criteria in the decision-making process.

The AHP has been instrumental in addressing complex nutritional challenges. For example, a systematic review of food recommender systems for diabetic patients highlighted the use of AHP to tailor dietary recommendations, considering individual preferences and nutritional needs, which is crucial for managing diabetes effectively [22]. AHP’s ease of use and ability to incorporate both qualitative and quantitative data have contributed to its successful application in various nutritional contexts, such as prioritizing dietary guidelines, evaluating food choices, and developing personalized meal plans [23,24]. Despite its widespread use, AHP’s effectiveness can be compromised by inconsistencies in pairwise comparisons, which are inherent to the method due to its reliance on subjective judgments. These inconsistencies can lead to unreliable decision outcomes, particularly in complex scenarios like meal planning, where multiple criteria need to be balanced.

Several approaches have been proposed to address the inconsistency issue in AHP. Eigenvalue-based methods are commonly used—such as Saaty’s consistency ratio (CR) [2]—which quantify the degree of inconsistency in a pairwise comparison matrix. While simple to implement, they may not always accurately reflect the actual level of inconsistency, especially in complex decision problems [25,26,27]. Optimization techniques are another approach, aiming to minimize the inconsistency of the comparison matrix by adjusting the pairwise judgments. Examples include goal programming and least squares methods [28]; however, these methods can be computationally expensive and may not always find the optimal solution. Heuristic algorithms, such as genetic algorithms and simulated annealing, offer a flexible and efficient way to search for consistent matrices [29,30]. They can handle complex decision problems and often find good solutions quickly. However, they may not guarantee finding the optimal solution and can be sensitive to parameter settings.

Our proposed PSO-SA hybrid algorithm falls under the category of heuristic algorithms. It aims to address the limitations of existing methods by combining the strengths of PSO’s global search capabilities with SA’s ability to escape local optima. This approach seeks to achieve both consistency and accuracy in pairwise comparisons, thereby enhancing the reliability and effectiveness of AHP-based meal-planning decisions.

Despite significant advancements in meal-planning optimization, many existing methods struggle to balance the complexity of multi-criteria decision-making with consistency and efficiency in results. Our proposed PSO-SA hybrid algorithm aims to address this gap by combining the strengths of PSO and SA, offering a novel approach to achieving consistent and accurate pairwise comparisons in AHP-based meal planning. This innovation enables more reliable and personalized nutritional recommendations, enhancing decision-making in complex dietary scenarios.

## 3. Methodology

To address the challenge of inconsistency in AHP pairwise comparisons, particularly in the context of complex nutritional decision-making like meal planning, we propose a hybrid algorithm that synergizes PSO and SA. This approach leverages the strengths of both algorithms to efficiently refine the initial, potentially inconsistent weight matrix into a consistent one that closely reflects the expert’s (nutritionist’s) judgments.

### 3.1. Analytic Hierarchy Process in the Context of Meal Planning

The Analytic Hierarchy Process (AHP) is an MCDM tool that assists experts in structuring complex decisions. It enables nutritionists to systematically break down meal planning into a hierarchical model, facilitating the prioritization of various criteria and alternatives through pairwise comparisons.

#### 3.1.1. Criteria and Constraints in Meal Planning

Drawing from our previous work [11], we recognize that meal planning for individuals, particularly those with dietary restrictions like diabetes, necessitates a comprehensive consideration of diverse factors. These encompass the following:

- Health and medication restrictions: dietary needs based on medical conditions, allergies, or medication interactions;
- Cultural and religious restrictions: food preferences and avoidances rooted in cultural or religious beliefs;
- Food availability: access to specific ingredients or cuisines based on location or seasonality;
- Budget limitations: affordability of meal options;
- Time constraints: preparation and cooking time available to the individual;
- Flavor preferences: taste preferences and dislikes;
- Popularity and ratings: consideration of popular or highly rated recipes;
- Serving size preferences: portion control and desired meal sizes.

#### 3.1.2. Weighting and Integration

Within the AHP framework, these criteria are organized hierarchically, and pairwise comparisons are conducted to establish their relative importance. The resulting weight matrix reflects the priority assigned to each criterion, guiding the subsequent evaluation and selection of meal options. To accurately represent individual needs, user surveys or direct input can be utilized to elicit preferences and assign weights to these criteria. This allows for a truly personalized meal-planning experience.

Central to AHP is the “nine value” scale introduced by Saaty (2001) [3], which assigns numerical values to pairwise comparisons to express the relative importance of one element over another. These values range from 1 (equal importance) to 9 (extreme importance), as shown in Table 1.

The AHP can be applied in three steps: (1) defining the vector of criteria weights; (2) computing the option scores matrix; and (3) grading the options.

The pairwise comparison *A* = {$a_{ij}$} is a square matrix *N* × *N*, where *N* is the number of criteria and $a_{ij}$ of matrix *A* represents the importance of the i-th criterion with respect to the *j*-th criterion based on Table 1. If the paired comparison is consistent, the values of the original upper diameter are inversely proportional to the values of the original diameter, and the main diameter is one.

(1) $$a_{ij}=\frac{1}{a_{ji}}\;\forall i\neq j,$$

$$a_{ii}=1$$

The 1 × *N* normalized eigenvector is obtained based on comparison matrices. Sum each column of *A* and normalize by dividing each matrix element by the column sum. Averaging across rows yields the normalized principal eigenvector:

(2) $$w_{i}=\frac{\sum_{i=1}^{N}{\bar{a}}_{ij}}{N}$$

where $w_{i}$ is the weight of the *i*-th criterion; and ${\bar{a}}_{ij}$ is the normalized value of the *j*-th element of the *i*-th row of matrix *A*.

An *M* × *N* matrix (where *M* is the number of alternatives and *N* is the number of criteria) is built as an option score matrix $S=\left\{s_{ij}\right\}$, where $s_{ij}$ represents the score of the i-th option with respect to the j-th criterion. To attain such scores, for each of the N criteria, a pairwise comparison matrix $p^{i}$, i = {1, …, N} is created. The matrix $P^{i}\;$ is a square matrix *M* × *M* as ${p^{i}=\{p}_{jk}^{i}\}$ where *M* is the number of options and $p_{jk}^{i}\;$ is the importance of the *j*-th option for the *k*-th option based on the *i*-th criterion. The constraint of matrix $P^{i}\;$ is the same as matrix *A*. Score vector is obtained for options based on each criterion, like the weight vector, and finally, the score matrix is attained as $P=[P^{1}\ldots \;P^{N}$]. In the final step, the ranked options vector v can be calculated by multiplying *P* and *w*.

(3) v=P·w

The maximum value $v_{i}$ shows the most desirable option.

#### 3.1.3. Consistency Rate

For an *N* × *N* square matrix *A* and eigenvector *w*, we calculate the following:

(4) Aw=λw

where *λ* is the eigenvalue. The largest eigenvalue is called the principal eigenvalue ${\;\lambda }_{max}$.

Saaty [31] has shown that for a consistent pairwise matrix, $\lambda_{max}$ is equal to the number of comparisons or ${\;\lambda }_{max}$ = *N*. Also, for all comparisons, $a_{ij}$, the transitivity rule is considered (Equation (5)):

(5) $$a_{ij}=a_{ik}.a_{kj}$$

Due to the 9-value scale limitation, Equation (5) is often violated. This is because when the options are between 1 and 9, their multiplication will likely exceed 9. It is hard to achieve complete consistency. To calculate the inconsistency rate, a measure is introduced as follows:

(6) $$CI=\frac{\lambda_{max}-N}{N-1}$$

We obtain the compatibility rate from Equation (7).

(7) $$CR=\frac{CI}{RI}$$

In Equation (7), *RI* is the Random Index, which Saaty [2] determines to estimate the expected consistency index for a randomly generated pairwise comparison matrix. The *RI* varies based on the number of elements being compared, as shown in Table 2.

The decision-maker should review the judgments if the consistency rate is greater than 0.1. Therefore, if we use AHP to determine the weights and the weights matrix is inconsistent, then the inconsistency must be resolved. Finding a consistent matrix would be time-consuming when numerous options and criteria exist.

#### 3.1.4. Objective Function in Meal Planning

In the context of meal planning, the objective is to discover a refined pairwise comparison matrix (representing the relative importance of different meal-planning criteria) that exhibits both consistency and fidelity to the nutritionist’s initial judgments.

- Consistency: The refined matrix should adhere to the transitivity rule of AHP, ensuring logical coherence in the prioritization of criteria. This is crucial for generating reliable and meaningful meal recommendations;
- Fidelity: the refined matrix should remain as close as possible to the original matrix, preserving the essence of the nutritionist’s expert opinion and the client’s expressed preferences.

To achieve these dual objectives, we define an objective function that quantifies the discrepancy between the refined matrix (*M′*) and the original matrix (*M*) and the degree of consistency in the refined matrix.

- Discrepancy Measure (*DI*): We adopt the distance scale introduced in [9] (Equation (8)) to calculate the distance between the alternative matrix (*M′*) and the initial matrix (*M*). In this context, *G* and *G′* represent row vectors containing the lower triangular elements of the original and refined pairwise comparison matrices, respectively. The division “./” is performed element-wise. A *DI* value of zero indicates perfect agreement between the two matrices.

(8) $$DI=\left|\left|G^{\prime }-G\right|\right|=\frac{\left|G^{\prime }./G\right|+\left|G./G^{\prime }\right|}{n^{2}-n}-1$$

- Consistency Measure (*λmax-N*): The difference between the largest eigenvalue (*λmax*) of the refined matrix and the number of criteria (*N*) serves as a measure of consistency. A smaller difference signifies better consistency;
- Combined Objective Function/Objective Index (*OI*): these two measures are integrated into a single objective function (Equation (9)).

(9) $$OI=DI+{\left(\lambda_{max}\right)}^{-n}$$

- The algorithm aims to minimize this objective function, thereby finding a refined matrix that is both consistent and faithful to the original expert judgments.

By optimizing this objective function, the proposed algorithm helps nutritionists navigate the complex decision-making landscape of meal planning, ensuring that the generated recommendations are not only scientifically sound and personalized but also respect the nuanced priorities and preferences of both the expert and the client.

### 3.2. The PSO-SA Hybrid Algorithm for Meal Planning

#### 3.2.1. Overview

Our hybrid PSO-SA algorithm is motivated by the need for a robust and efficient method to resolve inconsistencies in AHP pairwise comparison matrices, a common issue that can hinder the effectiveness of the AHP in real-world applications. We aim to achieve a balance between preserving the nutritionist’s original preferences and ensuring the logical consistency of the decision matrix.

The central idea is to utilize PSO to explore the solution space and quickly identify promising regions of consistent matrices. Then, SA is employed to refine the search in these promising regions, effectively escaping local optima and converging towards a globally optimal solution.

#### 3.2.2. Particle Swarm Optimization

Introduced in 1995, Particle Swarm Optimization (PSO) is an algorithm inspired by the social behaviors of organisms within large groups, such as flocks of birds or colonies of bees [32]. The core concept of PSO is to simulate a ”swarm” of particles moving through a multi-dimensional search space. Each particle adjusts its trajectory based on its own experience and the collective wisdom of the swarm. This dynamic adjustment guides the particles toward optimal solutions over successive iterations.

In PSO, each particle is essentially a point in an *n*-dimensional space, represented as $X_{i}(x_{i_{1}};x_{i_{2}},\ldots ,x_{i_{n}})$. The algorithm updates each particle’s position based on two key values: the best solution it has encountered, known as (*Pbest*); and the best solution found by any particle in the swarm, known as (*Gbest*).

The simplicity of PSO lies in its reliance on only two equations to update the velocity and position of the particles:

(10) $$\begin{array}{cc} Velocity_{i}\left(t+1\right) & =w\times Velocity_{i}\left(t\right)\\ & +c_{1}\times random()\times \left(P_{best}\left(t\right)-Position_{i}\left(t\right)\right)\\ & +c_{2}\times random()\times (G_{best}\left(t\right)-Position_{i}\left(t\right)) \end{array}$$

(11) $$Position_{i}(t+1)=Position_{i}(t)+Velocity_{i}(t+1)$$

where $Position_{i}$ and $Velocity_{i}$ represent the current position and velocity of the particle, respectively; the function random() generates a uniform random number between 0 and 1; the coefficients $c_{1}$ and $c_{2}$, the cognitive and social scaling factors, are typically set to 2; and *w* is the inertia weight, which moderates the particle’s velocity to balance exploration and exploitation.

To prevent the particles from diverging too far from the search space, the velocity is constrained by a maximum value $V_{imax}.$ If the calculated velocity exceeds $V_{imax}$, it is capped at $\pm V_{imax}$, ensuring that the particles’ movements remain within a controlled range.

#### 3.2.3. Simulated Annealing

Simulated Annealing (SA) is a heuristic optimization technique that was first introduced in 1983, drawing inspiration from the process of annealing in metallurgy [33]. This probabilistic technique is renowned for its ability to escape local optima, making it a valuable tool in complex optimization scenarios. The SA algorithm mimics the physical process where a material is heated and then slowly cooled to minimize defects and achieve a stable crystal structure.

The SA algorithm begins with a randomized initial solution and introduces small, random changes to this solution at each step. The objective function value of the new solution, $f\left(s_{n}\right)$, is then compared to the current solution, $f(s_{c})$, as shown in Equation (12):

(12) $$∆E=f\left(s_{n}\right)-f(s_{c})$$

The decision to accept the new solution is governed by a probability function, detailed in Equation (13):

(13) $$p=\left\{\begin{array}{c} 1\;if\;∆E<0\\ e^{\frac{∆E}{T}}\;otherwise \end{array}\right.$$

In this context, ∆E represents the change in the objective function value, and *T* is a temperature parameter that gradually decreases over time according to Equation (14):

(14) $$T_{i+1}=\gamma T_{i}$$

where γ is a factor between zero and one, dictating the rate at which the temperature decreases. Initially, SA allows a higher probability of accepting worse solutions to facilitate exploration and prevent premature convergence to local optima. As the algorithm progresses and the ”temperature” lowers, accepting suboptimal solutions becomes less likely, steering the search towards the global optimum.

The effectiveness of SA is partially dependent on the quality of the initial solution and the cooling schedule. The algorithm concludes either upon finding an optimal solution or after a pre-determined number of iterations, as described by Bertsimas in 1993 [34].

#### 3.2.4. PSO-SA Hybrid Algorithm

##### Integration of PSO and SA

The PSO-SA hybrid algorithm [35,36] stands as a testament to the power of combining different optimization strategies to achieve superior results. It represents an innovative fusion of Particle Swarm Optimization (PSO) and Simulated Annealing (SA), capitalizing on the strengths of both to overcome their respective limitations. PSO is known for its robust search capabilities and rapid convergence, while SA excels in local search and escaping local optima. This amalgamation leverages the exploratory prowess of PSO with the exploitative finesse of SA, creating a comprehensive search strategy that is both wide-ranging and detail-oriented.

In the context of meal planning, the PSO-SA algorithm refines the AHP weight matrix, which reflects the relative importance of various criteria (e.g., glycemic control, nutrient density, palatability, and convenience). This refined matrix guides the evaluation and scoring of meal options, ensuring that the final recommendations align closely with both the nutritionist’s expertise and the client’s individual needs and preferences. In this hybrid model, PSO quickly navigates the search space, identifying regions of potential optimality. Upon finding a promising solution, SA takes over, conducting an intensive local search in the vicinity of PSO’s best-found solution. This dual-phase approach ensures that the algorithm does not prematurely converge on suboptimal solutions, a common pitfall in optimization algorithms.

The algorithm’s inherent random movement is key to its success as it allows for exploration of the search space without being confined to a deterministic path. The PSO component propels the algorithm towards areas of interest, while the SA meticulously refines the search, honing in on the optimal solution.

To address the challenge of finding a consistent matrix that also reflects an expert’s initial suggestions, we propose a hybrid algorithm that utilizes the strengths of PSO and SA. The algorithm aims to refine the pairwise weight matrix to achieve consistency while remaining as close as possible to the original matrix.

The algorithm initiates by generating random sets of meal options (particles), evaluates them using the AHP-based objective function (considering the refined weight matrix), and iteratively updates these meal combinations based on their individual best scores (*Pbest*) and the overall best score (*Gbest*). If the *Gbest* surpasses a pre-defined threshold, new meal combinations are generated in its vicinity, and the process continues until convergence or the maximum number of iterations is reached. The final *Gbest* represents the optimal meal plan that best satisfies the client’s diverse criteria and constraints.

After updating all particles within a generation, if the obtained *Gbest* exceeds a pre-defined error threshold, new particles are introduced within the neighborhood of (*Gbest*). The size of this neighborhood decreases with each iteration. The evaluation function is then recalculated for these new particles, and if any demonstrate a value better than the current (*Gbest*), the (*Gbest*) is updated to this new value. In cases where no improvement is found, the difference in evaluation ∆E is computed, and a new (*Gbest*) based on a probability function is potentially accepted. Subsequently, the velocity and position of each particle are updated in preparation for the next generation. This process is iterated until the algorithm either reaches the maximum number of iterations or meets the specified error criteria.

In developing this proposed algorithm, we considered the need for a method that balances global and local search capabilities, given the complexity of the optimization problems we aimed to solve. The rationale behind combining PSO and SA is rooted in the complementary strengths of these techniques: PSO’s ability to swiftly explore the search space and SA’s proficiency in refining solutions to escape local optima. This synergy is particularly advantageous in refining the pairwise weight matrix, ensuring both consistency and adherence to expert suggestions.

The parameters for our hybrid algorithm, including the inertia weight, cognitive and social coefficients for PSO, and the cooling schedule for SA, were carefully selected through empirical testing and domain expertise. These settings ensure a balanced trade-off between exploration and exploitation, which is crucial for the algorithm’s performance.

In Algorithm 1, we introduce our proposed algorithm, detailing our approach’s main steps and procedures. This pseudocode offers a comprehensive overview of the algorithm’s structure and workflow.
**Algorithm 1**. PSO-SA Algorithmitr = 1  Initialize swarm size, T,ᾳ  Initialize particle Position and Velocity  Stop Condition = maxiterations or predefine error  while Not stop Condition do    for each particle I = 1 to swarm size, do       Evaluate f(particle(i))     if the f(particle(i)) is better than the f(Pbest) then        Update current Pbest.    end    if f(Pbest) is better than f(Gbest) then       Gbest = Pbest    end  end  if f(Gbest) > predefine error then    Generate neighborhoods(Gbest, ᾳ)    For j = 1 to neighborhoods size do       Evaluate f(Neighborhood(j))       if the f(Neighborhood(j)) is better than the f(Gbest) then      Gbest = Neighborhood(j)       Elseaccept the Neighborhood with a probability *p* defined by      ∆E = f(Neighborhood(i).Pbest) − f(Gbest)      P = e^−∆E/T^       end       update T   end  update ᾳ  itr = itr + 1 endupdate ᾳ itr = itr + 1 end

### 3.3. Advanced Decision-Making Tool in Nutritional Counseling

The practical application of our algorithm is demonstrated through its ability to refine the pairwise weight matrix to achieve consistency while remaining as close as possible to the original matrix. This refined matrix is then used by nutritionists to evaluate and score meal options, enabling them to recommend meals that optimally align with the client’s needs and preferences.

By incorporating the PSO-SA hybrid algorithm into their practice, nutritionists can be equipped with an advanced decision-making tool that enhances the accuracy and personalization of meal planning. This methodology empowers nutritionists to deliver dietary recommendations that are scientifically sound and tailored to their client’s unique preferences and health goals.

## 4. Evaluations

To assess the efficacy and real-world applicability of the PSO-SA hybrid algorithm, we employed a multi-faceted evaluation approach encompassing the following.

### 4.1. Prototype System and User Interaction

**Figure 1:**
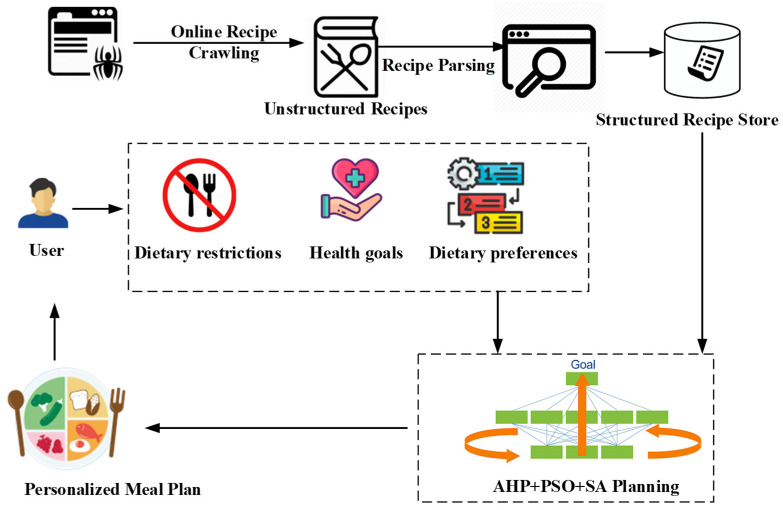
The architecture of the meal planning app using the proposed algorithm.

We have integrated the PSO-SA hybrid algorithm into a previously developed mobile app designed for personalized meal planning [4]. As shown in Figure 1, this app streamlines the meal-planning process by leveraging a web crawler to gather diverse recipes, a recipe parser to extract key nutritional information, and a sophisticated meal-planning module powered by the PSO-SA algorithm.

User experience: Users begin by creating a profile and inputting their health conditions, dietary restrictions, preferences, and goals. The app then utilizes the PSO-SA algorithm to generate personalized meal plans that cater to these individual needs. Users can further interact with the app by providing feedback on recommended meals, allowing for continuous refinement and adaptation of the meal plans.

### 4.2. Use Case Evaluations

We present two illustrative use cases that mirror real-world scenarios encountered by nutritionists. These cases showcase the algorithm’s capacity to optimize meal planning for individuals with distinct dietary requirements and health objectives.

**Use case 1—Personalized Meal Planning for a Client with Type 2 Diabetes**

Client 1’s profile is as follows:
  
**Client1 Profile**
45-year-old male recently diagnosed with type 2 diabetes;BMI of 30 (classified as obese);Sedentary lifestyle;Dietary preferences: enjoys Mediterranean cuisine, prefers home-cooked meals, dislikes overly bland food;Dietary restrictions: limiting carbohydrate intake, saturated fats, and added sugars is necessary;Health goals: improve glycemic control (HbA1c levels), manage weight (lose 5% body weight in 3 months), increase energy levels.

**Pairwise Comparison Matrix and Refinement:**

The nutritionist collaborates with the client to identify the following criteria as crucial for meal selection:

- Glycemic control (GC);
- Nutrient density (ND);
- Palatability (P);
- Convenience (C).

The initial pairwise comparison matrix, based on the client’s priorities and the nutritionist’s expertise, is presented in Table 3. This matrix reveals a strong emphasis on glycemic control, but its consistency ratio (CR) of 0.12 exceeds the acceptable threshold, indicating inconsistency in the judgments.

Applying the PSO-SA hybrid algorithm refines this matrix, balancing the need for consistency with the client’s preferences. The resulting refined matrix (Table 4) achieves a CR of 0.017, signifying acceptable consistency while retaining the prioritization of glycemic control.

**Meal Evaluation and Recommendation:**

Using the refined matrix, the nutritionist evaluates various meal options. For instance, a typical Mediterranean lunch option like grilled chicken salad is scored against the following criteria:

- GC score: high (due to low glycemic index ingredients);
- ND score: high (rich in vitamins and minerals);
- P score: moderate (flavorful with herbs and spices);
- C score: moderate (requires preparation but can be made in advance).

The algorithm calculates weighted scores for each meal, factoring in the relative importance of each criterion as defined in the refined matrix. This enables the nutritionist to recommend a weekly meal plan comprising diverse Mediterranean dishes that align with the client’s health goals and dietary preferences, promoting improved glycemic control and weight management.

**Use case 2—Tailoring Dietary Recommendations for an Elderly Client with Chronic Conditions**

Client 2’s profile is outlined below:
  
**Client2 Profile**
70-year-old female with a history of hypertension and osteoporosis;BMI of 22 (within the normal range);Moderately active lifestyle with daily walks and light exercise;Dietary preferences: prefers a plant-based diet, enjoys mild flavors, and has a penchant for traditional dishes;Dietary restrictions: needs to limit sodium intake and increase calcium-rich foods to manage hypertension and support bone health;Health goals: maintain blood pressure within the normal range, prevent bone density loss, and enhance overall well-being.

**Pairwise Comparison Matrix and Refinement:**

The nutritionist collaborates with the client to prioritize the following criteria for meal selection:

- Blood pressure management (BPM);
- Bone health (BH);
- Flavor (F);
- Ease of preparation (EP).

The initial pairwise comparison matrix (Table 5) emphasizes blood pressure management but shows inconsistency with a CR of 0.14.

Applying the PSO-SA hybrid algorithm leads to a refined matrix (Table 6) with a CR of 0.015, ensuring consistency while preserving the client’s preferences.

**Meal Evaluation and Recommendation:**

The refined matrix guides the evaluation of meal options like kale and quinoa salad:

- BPM score: high (low in sodium);
- BH score: high (rich in calcium);
- F score: moderate (mild yet flavorful);
- EP score: high (easy to prepare).

Leveraging the algorithm’s weighted scoring, the nutritionist recommends a personalized meal plan featuring plant-based, calcium-rich, low-sodium dishes that cater to the client’s health goals and dietary needs.

**Overall Impact:**

These use cases highlight the PSO-SA hybrid algorithm’s role in enhancing personalization and addressing inconsistencies in dietary interventions. By integrating client data and preferences into the AHP framework, the algorithm empowers nutritionists to make informed decisions, leading to improved client adherence and satisfaction. Its effectiveness underscores its potential as a valuable tool in nutritional counseling.

### 4.3. Additional Evaluation Approaches

To further validate the effectiveness of the PSO-SA hybrid algorithm, we employed additional evaluation methods beyond the use case demonstrations, as outlined below.

#### 4.3.1. Algorithm Performance Metrics

**Figure 2:**
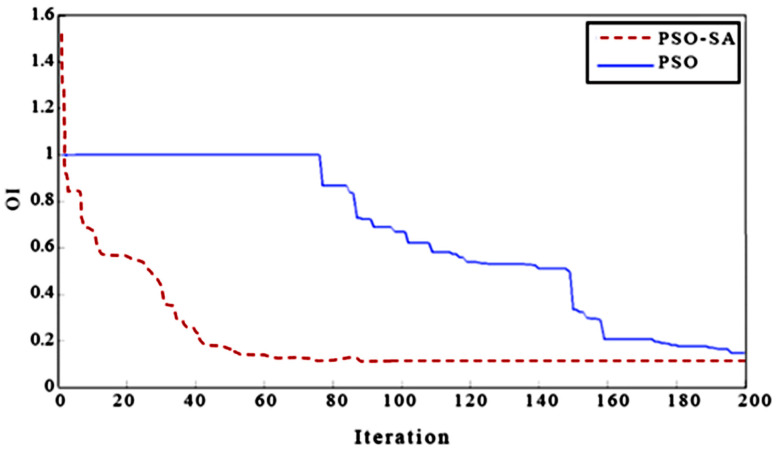
The comparison of the results of the PSO-SA and PSO.

We quantitatively assessed the algorithm’s performance using the following metrics (Table 7):

Figure 2 provides a visual comparison of the proposed PSO-SA implementation and the standard PSO algorithm in terms of the objective function value (OI) over iterations. It illustrates that PSO-SA consistently achieves a lower OI (indicating a better solution) compared to PSO, demonstrating its superior performance in finding consistent matrices that closely align with the nutritionist’s initial judgments.

#### 4.3.2. Manual Verification

All meal plans generated by the PSO-SA algorithm underwent a rigorous manual verification process. A team of researchers meticulously reviewed each meal plan to ensure the following:

- Adherence to dietary guidelines: the meal plans complied with established dietary guidelines for the clients’ specific health conditions and dietary restrictions;
- Nutritional adequacy: the meal plans provided a balanced and sufficient intake of essential nutrients, considering the clients’ age, gender, activity levels, and health goals;
- Appropriateness for client profiles: the meal plans reflected the clients’ individual preferences, cultural considerations, and lifestyle factors, promoting adherence and satisfaction.

The results of this manual verification process are summarized in Table 8.

The 98.09% approval rate across 30 use cases underscores the PSO-SA algorithm’s ability to generate meal plans that are not only personalized and consistent but also nutritionally sound and clinically appropriate.

These multi-faceted evaluations collectively demonstrate the practicality and effectiveness of the PSO-SA hybrid algorithm in real-world nutritional counseling scenarios. The algorithm’s capacity to refine AHP decision-making, coupled with its integration into a user-friendly mobile app, positions it as a valuable tool for nutritionists seeking to provide personalized and evidence-based dietary recommendations.

## 5. Limitations and Future Directions

While the PSO-SA hybrid algorithm shows promise in refining the AHP process for personalized meal planning, it is not without limitations. One such limitation is the potential for premature convergence on suboptimal solutions if the balance between the PSO and SA components is not carefully managed. Additionally, the algorithm’s performance is highly dependent on the parameter settings, which may require fine-tuning for different use cases.

Another limitation is that the algorithm relies on user input to define dietary preferences, restrictions, and health goals. The accuracy and consistency of these inputs directly impact the quality of the generated meal plans. Furthermore, the current implementation focuses on meal planning for individuals with specific dietary needs, such as diabetes. Further research is needed to explore its applicability to other populations and dietary scenarios.

Future research should aim to address these limitations. This could involve investigating alternative techniques to mitigate premature convergence and exploring adaptive parameter tuning mechanisms to enhance the algorithm’s robustness across different contexts. Additionally, incorporating more comprehensive user data, such as individual genetic predispositions or gut microbiome profiles, could further personalize meal-planning recommendations. Integrating real-time feedback mechanisms within the mobile app could also enable dynamic adjustments to meal plans based on user experience and outcomes.

## 6. Discussion

Our research introduces the PSO-SA hybrid algorithm to optimize consistency in AHP-based meal planning. This approach builds on previous efforts to enhance the AHP method and streamline personalized nutrition through various techniques. Our hybrid algorithm offers improvements in balancing global and local search optimization while minimizing consistency ratios.

To demonstrate the advantages of the PSO-SA hybrid approach, we compared our findings with existing studies, focusing on meal planning and consistency in decision-making frameworks. Table 9 presents a summary of key aspects, including the study focus, optimization techniques used, the prioritization of preferences, efforts to minimize the consistency ratio, and the balance between global and local searches. This comparative analysis highlights the comprehensive strengths of our PSO-SA hybrid algorithm, particularly its ability to maintain both global and local search efficiency while ensuring consistency in AHP-based decision-making processes.

## 7. Conclusions

The AHP framework has proven valuable for structuring complex decision-making processes like meal planning but maintaining consistency in pairwise comparisons becomes increasingly challenging as the number of criteria and options grows. This inconsistency can affect the quality of personalized meal plans, which must balance various factors such as glycemic control, nutrient density, and individual preferences. Our research addresses this challenge by integrating the PSO-SA hybrid algorithm into the AHP framework.

The PSO-SA hybrid algorithm plays a crucial role in resolving inconsistencies within the AHP framework by automating the refinement process of pairwise comparison matrices. This algorithm not only ensures that the final matrix aligns closely with expert judgment but also incorporates individual preferences more accurately, creating a more personalized and reliable meal-planning tool. In this way, the PSO-SA algorithm and the AHP framework work together to enhance both the consistency and effectiveness of decision-making in meal planning.

The different components of this work—AHP, PSO, and SA—are interconnected in that the PSO-SA hybrid improves the traditional AHP method by addressing its limitations. The introduction of heuristic optimization techniques allows for more accurate and consistent decisions in meal planning, ensuring that nutritionists can create tailored meal plans efficiently and effectively.

Moving forward, further development of this work could involve validating the PSO-SA hybrid algorithm in real-world settings through clinical trials and user studies. This will help assess its impact on adherence to dietary recommendations and client satisfaction. Additionally, future research could explore integrating advanced machine learning techniques to expand the algorithm’s capabilities, allowing it to account for a wider range of factors in real time. Such developments could revolutionize personalized meal planning, enabling nutritionists to generate meal plans that dynamically adapt to individual health conditions and preferences.

## Figures and Tables

**Table 1:** The “9 values” scale [3].

Definition	Intensity of Importance
1	Equal importance
2	Weak importance
3	Moderate importance
4	Moderate plus
5	Strong importance
6	Strong plus
7	Very strong or demonstrated importance
8	Very, very strong
9	Extreme importance

**Table 2:** The random indicator.

n	3	4	5	6	7	8	9	10
RI	0.58	0.9	1.12	1.24	1.32	1.41	1.45	1.49

**Table 3:** The initial pairwise comparison matrix.

	GC	ND	P	C
GC	1	6	8	9
ND	1/6	1	3	5
P	1/8	1/3	1	4
C	1/9	1/5	1/4	1

**Table 4:** The refined pairwise comparison matrix.

	GC	ND	P	C
GC	1	4	6	8
ND	1/4	1	2	4
P	1/6	1/2	1	2
C	1/8	1/4	1/2	1

**Table 5:** The initial pairwise comparison matrix for client 2.

	BPM	BH	F	EP
BPM	1	6	8	9
BH	1/6	1	4	6
F	1/8	1/4	1	4
EP	1/9	1/6	1/4	1

**Table 6:** The refined pairwise comparison matrix for client 2.

	BPM	BH	F	EP
BPM	1	3	6	8
BH	1/3	1	3	4
F	1/6	1/3	1	2
EP	1/8	1/4	1/2	1

**Table 7:** Performance metrics.

Metric	Description
Consistency Improvement	The reduction in Consistency Ratio (CR) after applying PSO-SA demonstrating its effectiveness in resolving inconsistencies.
Convergence Speed	The number of iterations required for the algorithm to converge to a consistent matrix showcasing its efficiency.
Solution Quality	Comparison of the final objective function value achieved by PSO-SA against standard PSO, highlighting its ability to find superior solutions.

**Table 8:** Manual verification summary.

Number of Use Cases	Number of Meal Plans Generated	Number of Meal Plans Approved	Approval Rate
30	30 × 7 (one week for each client)	206	98.09%

**Table 9:** Comparison of studies on meal planning and AHP. The “✓” indicates that the criterion is addressed in the study, while “✗” indicates that it is not addressed.

Study	Focus	Optimization Techniques	Preference Priority	Minimize Consistency Ratio	Global and Local Search Balance
Amiri et al. [10]	Meal Planning	Reinforcement Learning and Collaborative Filtering	✓	✗	✗
Othman et al. [12]	Meal Planning	Collaborative Filtering	✓	✗	✗
Benítez et al. [6]	AHP	Matrix Minimization	✗	✓	✗
Zadeh et al. [4]	Meal Planning	MCDM Approach	✓	✗	✗
Lin et al. [5]	AHP	Adaptive AHP	✗	✓	✗
PSO-SA Hybrid Approach	Combination (Meal Planning and AHP)	PSO-SA Hybrid Algorithm	✓	✓	✓

## Data Availability

The original contributions presented in the study are included in the article, further inquiries can be directed to the corresponding author/s.
